# Pathological features of African horse sickness virus infection in IFNAR^−/−^ mice

**DOI:** 10.3389/fvets.2023.1114240

**Published:** 2023-03-30

**Authors:** Luke M. Jones, Phillippa C. Hawes, Francisco J. Salguero, Javier Castillo-Olivares

**Affiliations:** ^1^The Pirbright Institute, Woking, United Kingdom; ^2^United Kingdom Health Security Agency, UKHSA-Porton Down, Salisbury, United Kingdom; ^3^School of Veterinary Medicine, University of Surrey, Guildford, United Kingdom; ^4^Laboratory of Viral Zoonotics, Department of Veterinary Medicine, University of Cambridge, Cambridge, United Kingdom

**Keywords:** African horse sickness, arbovirus, immunofluorescence, immunohistochemistry, mouse model, pathology

## Abstract

African Horse Sickness (AHS) is a vector-borne viral disease of equids. The disease can be highly lethal with mortality rates of up to 90% in non-immune equine populations. The clinical presentation in the equine host varies, but the pathogenesis underlying this variation remains incompletely understood. Various small animal models of AHS have been developed over the years to overcome the financial, bio-safety and logistical constraints of studying the pathology of this disease in the target species. One of the most successful small animal models is based on the use of interferon-alpha gene knock-out (IFNAR^−/−^) mice. In order to increase our understanding of African Horse Sickness virus (AHSV) pathogenesis, we characterised the pathology lesions of AHSV infection in IFNAR^−/−^ mice using a strain of AHSV serotype 4 (AHSV-4). We found AHSV-4 infection was correlated with lesions in various organs; necrosis in the spleen and lymphoid tissues, inflammatory infiltration in the liver and brain, and pneumonia. Significant viral antigen staining was only detected in the spleen and brain, however. Together these results confirm the value of the IFNAR^−/−^ mouse model for the study of the immuno-biology of AHSV infections in this particular *in vivo* system, and its usefulness for evaluating protective efficacy of candidate vaccines in preclinical studies.

## 1. Introduction

African Horse Sickness Virus (AHSV) is a member of the *Orbivirus* genus, within the family *Reoviridae*. It is endemic throughout parts of sub-Saharan Africa, although outbreaks have occurred in northern Africa and southern Europe (1–3). There is currently no treatment for the disease. The disease is vector-borne, being spread by the bites of *Culicoides sp*. midges, particularly *Culicoides imicola* (4, 5). Although in horses the disease is highly lethal, the morbidity and mortality are lower in donkeys and mules. In zebras the disease is asymptomatic, manifesting only as febrile syndrome (6). However, AHSV pathogenesis is not fully understood. Some studies have shown that certain proteins are linked to a virulent phenotype (7), and that the outcome of infection is highly dependent on viral factors (8).

The whole viral genome is approximately 18.5kb in length (9, 10) and is composed of ten segments of double-stranded RNA, enclosed within a triple layer capsid. The inner core of this capsid is formed by VP3, which surrounds the genome and transcription complex proteins (VP1, VP6, and VP4). The outer core is formed by a layer of VP7 trimers and the outer capsid layer is comprised of two trimeric proteins, VP2 and VP5 (11). Six viral non-structural (NS1, NS2, NS3, NS3a, NS4-I, and NS4-II) proteins are expressed during replication. There are nine different serotypes of AHSV and serotype is determined by the VP2 protein (12).

AHSV can infect a range of cell types within the equine host, particularly endothelial cells (13, 14) and specific leucocytes, especially monocyte-macrophages (13, 15). Infection often induces pathology in the lungs, heart, liver and spleen, and viral replication occurs mainly within the endothelial cells of blood vessels supplying these organs (13, 16). The virus also shows an ability to infect immune cells and within the lung AHSV seems to infect pulmonary intravascular macrophages (15). The virus has also been detected in association with monocytes and possibly lymphocytes (17).

There are four types of clinical presentations of AHS in the equine host: the pulmonary form, the cardiac form, the mixed form and the febrile form (18). The pulmonary form is characterised by respiratory distress, a short incubation time of 3 to 5 days, and extremely high mortality (near 100%). The cardiac form is the second most severe manifestation, with an incubation period of < 14 days (18) and a characteristic subcutaneous oedema (14), particularly around the head and neck with a characteristic swelling of supraorbital fossae. Mortality for this form of disease ranges from 50 to 70%. The febrile form is the mildest form of disease, with variable incubation time (4 to 14 days), mild symptoms and no mortality. The mixed form combines clinical signs from the cardiac and pulmonary forms, as well as having roughly intermediate incubation periods and mortality rates. It is currently unknown whether the different disease presentations are distinct or part of a continuous spectrum of the same pathological process. Some researchers suggest these differences are in large part due to viral factors, and little to do with differences within the equine host (8). One study indicates that the pulmonary and cardiac forms of disease are linked to a specific viral tropism for the heart and lung respectively, whilst the fever form of disease is associated with replication in the spleen (16). Other experiments have shown that virus strain was the determining factor on the outcome of infection when randomised groups of horses were challenged with AHSV (8). A study involving the use of recombinant viruses established that certain proteins play a particularly key role in pathogenicity; the two outer capsid proteins, VP2 and VP5, as well as NS3/NS3a (7). One recent study indicates that pathogenesis may also be linked to the dysfunctional immune response of the host to infection, influenced by the viral ability to modulate apoptosis in infected cells (19).

Vaccinology, pathogenicity and virulence studies of AHSV are difficult to perform in the target species for logistical, ethical and financial reasons. Therefore, various researchers have used small animal models to study aspects of vaccinology or virulence of AHSV (7, 20). IFNAR^−/−^ mice are a genetically engineered mouse strain that lacks a functional type 1 interferon (IFN) receptor, inhibiting their innate immune response and leaving them more susceptible to viral infection (21). They have previously been utilised as a small animal model for various viral diseases, including Ebola (22) and Zika (23). They have also been used to study the pathogenesis of Bluetongue Virus infection (24), a virus closely related to AHSV, and subsequently they have also been used to test the immunogenicity and protective efficacy of recombinant AHSV-VP2 based vaccines for AHSV and BTV (20, 25–29). In these experiments AHSV infection of IFNAR^−/−^ mice was shown to induce clinical signs, lethality, viraemia and lesions in various tissues. The presence of AHSV antigen was also detected in various tissues (20). We have extended this work and further characterised the pathological features of AHSV-4 infection in this small animal model.

## 2. Materials and methods

### 2.1. Viruses

The Madrid-89 strain of AHSV-4 virus was used for the challenge of IFNAR^−/−^ mice. The virus was initially isolated from an infected horse in Madrid in 1987 after 1 passage in mouse brain and 6 subsequent passages in BHK-21 cells (Pirbright reference collection number RSArah4/03). This virus was subsequently passaged twice BHK-21 cells. The titre of this stock was 10^6^ TCID_50_ / ml.

### 2.2. Mouse challenge

All experiments with live animals were carried out under the Home Office Animals (Scientific Procedures) Act (1986) regulations. Experiments were approved by the Animal Welfare and Ethical Review Board (AWERB) of The Pirbright Institute. Five IFNAR^−/−^ mice were allowed to acclimatise to biosafety level 3 facilities at the Pirbright Institute for 1 week before the experiment began. Mice were 6–8 weeks old when they were inoculated subcutaneously in the dorsal neck area with 2 × 10^5^ TCID_50_/mouse in a 50 μl volume. Through regular clinical observations, at least 4 times per day, any mice that reached humane endpoints were euthanised by an overdose of anaesthetic. Clinical observations were scored as described in Table 1.

**Table 1:** Scoring of clinical signs in AHSV-infected mice, and humane endpoints.

**Clinical sign of disease**	**Score indication**	
Ruffled Hair/Pilo-erection	0 = absent		1 = present
Separation from the group	0 = absent		1 = present
Reduced activity	0 = absent	1 = present	**2** **=** **frequent**
Hunching	0 = absent	1 = present	**2** **=** **frequent**, **>24 h**
Inflammation at injection site	0 = absent	1 = present	**2** **=** **present and painful**
Vocalisation	0 = absent	1 = present	**2** **=** **frequent**
Laboured breathing	0 = absent	1 = present	**2** **=** **frequent**
Abscess formation	0 = absent		**1** **=** **present**
Weight loss	0 = absent	1 = present	**2** **=** **>10%**
Dehydration	0 = absent		**1** **=** **present**
Periorbital swelling	0 = absent	1 = present	**2** **=** **Compromising vision**
Tremors	0 = absent		**1** **=** **present**
Ataxia	0 = absent		**1** **=** **present**
Paralysis or paresis	0 = absent		**1** **=** **present**
Circling gait	0 = absent		**1** **=** **present**
Ocular watery discharge	0 = absent		**1** **=** **present**
Nasal watery discharge	0 = absent		**1** **=** **present**

Scores that necessitated humane euthanisation are in bold.

Control (uninfected) mouse tissue, from mice with a C57/Bl6 genetic background, was obtained from the University of Surrey to act as a negative control in histopathology and immunohistochemistry runs.

### 2.3. Histopathology

At *post mortem*, the full carcass of every mouse was fixed by immersion in 10% buffered formalin at room temperature (RT), after opening the thoracic and abdominal cavities for exposure of internal organs for over a week. A *post mortem* examination was carried out with the fixed carcass following a standard protocol and samples from the following organs were removed: brain, heart, liver, spleen, kidney, large intestine, thymus, pancreas and a skin sample from the inoculation area. Immediately following dissection, samples were further fixed in 10% formalin and embedded into paraffin wax. Additional samples were preserved in 10% formalin for immunofluorescence.

Sectioning and haematoxylin and eosin (HE) staining were performed according to standard laboratory procedures at the University of Surrey Veterinary Pathology Centre using an automated stainer (Sakura Tissue-Tek DRS 2000). Briefly, 4 μm sections were moved to distilled water before nuclei were stained with haematoxylin. Acid-alcohol (0.3%) was then applied, before rinsing in running water. The eosin stain was applied for 2 min before dehydration and mounting. Stained organ sections were examined under the light microscope and histological lesions were recorded. Images were captured directly from the microscope (Nikon NI series) using *Nikon NIS-BR* software.

### 2.4. Immunohistochemistry

Four micrometre thick sections were cut from paraffin-wax tissue blocks, and the ABC Complex reagent method (Vector Laboratories, California, USA) was used for staining of CD3, CD45R, Ly6G, and F4/80 markers. Initial steps (dewaxing, endogenous peroxidase block and wash) were performed in an automated stainer (Sakura Tissue-Tek DRS 2000). Slides were then clipped to plastic mounts (Sequenza) and washed through twice with tris-buffered saline (TBS). Slides were then treated with proteinase K (PK) (diluted to 40 μl PK in 2 ml of TBS) for 10 min at room temperature, before being washed twice with TBS. Blocking serum (10% normal goat serum diluted in TBS) was then applied for 20 min, followed by primary antibody (Table 2). Samples were then incubated at 4°C for 20 h, before another two washes in TBS. The associated secondary antibody was then applied. After a 30 min incubation with secondary antibody, slides were washed twice in TBS, before a Vector Elite ABC conjugate was applied for 30 min. Another two washes in TBS were performed, followed by 10 min incubation with NOVA RED (Vector Laboratories; composed according to manufacturer's instructions). The NOVA RED reaction was halted with another double TBS wash. The final steps (wash in water, counterstain with haematoxylin, dehydration in ethanol, clear in xylene) were performed in a Sakura Tissue-Tek DRS 2000 automated stainer. Slides were mounted and coverslipped with DPX mountant. Appropriate positive and negative controls were used during each immunohistochemical run.

**Table 2:** List of antibodies and associated dilutions, immunohistochemistry.

**Cell marker (cell type)**	**Primary antibody, dilution (manufacturer), dilution**	**Epitope demasking step**	**Secondary antibody, dilution (manufacturer)**
CD3 (T cell)	Mouse anti-human CD3 (invitrogen), 1/500	PK enzyme, 30 min	Goat anti-mouse (1:200)
CD45R (B cell)	Rat anti-mouse CD45R (B220) (life technologies), 1/100	PK enzyme, 30 min	Goat anti-rat (1:200)
F4/80 (Macrophage)	Rat anti-mouse F4/80 (BIO-RAD), 1/100	PK enzyme, 30 min	Goat anti-rat (1:200)
Ly6-G (Neutrophil)	Rat anti-mouse Ly6-G (life technologies), 1/25	PK enzyme, 30 min	Goat anti-rat (1:200)

Image analysis was used to quantify the levels of IHC staining, by using the *NIS-Elements BR 64-bit* software, version 4.40.00 (Nikon, Japan). Briefly, to analyse the spleen images (3–4 per section) were taken at 40x magnification, sufficient to include the entire section, and the software manually calibrated to identify positive staining. The total quantity of positive staining within an image was identified, and then divided by the area of the spleen (calculated by the software after use of region-of-interest (ROI) tools). An average level of positive staining was calculated for each individual spleen from three or four images. In the brain, up to 20 lesions (perivascular cuffing) were imaged at x400, and again the software was manually calibrated to identify positive staining. An average level of positive staining for each cell type was recorded for each individual. The same approach was used to quantify positive staining in uninfected splenic tissues. Uninfected brain tissue contained no lesions.

### 2.5. Immunofluorescence

Formalin fixed “wet tissues” were cut in 70 μm thick sections using a vibrating microtome (Leica VT1000 S), treated with 0.1% Triton [diluted in phosphate buffered saline (PBS)] for 1 h, and 0.5% bovine serum albumin (BSA) in PBS overnight. Primary antibody was derived from rabbit polyclonal antisera specific for AHSV VP7 antigen, diluted 1/1000 in 0.5% BSA/PBS buffer and applied to the sections. After 90 min at RT samples were washed three times in PBS. Samples were then incubated with 1:200 fluorescein-conjugated anti-rabbit IgG (Alexa 488, ThermoFisher Scientific) for 90 min at room temperature. Three more washes in PBS were performed before a 30 min incubation with 4',6-diamidino-2-phenylindole (DAPI, ThermoFisher Scientific, 1:5000 in distilled water) and another three washes were performed before the final thirty-minute incubation with phalloidin (Life Technologies, 1:25 dilution in PBS). Sections were then mounted and imaged using a Leica SP8 confocal laser scanning microscope at 400x and 630x magnification, utilising *LasX* software.

### 2.6. Statistics

Statistical tests were performed with *GraphPad Prism* software, version 7.00. Tests utilised include an Ordinary one-way ANOVA, two-way ANOVA, and Tukey's multiple comparisons test.

## 3. Results

### 3.1. Clinical signs of infection and survival

Observed clinical signs of infection included ataxia, circling gait, ocular watery discharge, reduced activity, tremors and weight loss (Table 3). Two out of five mice were euthanised before the end of the experiment for humane reasons, on day 8 and day 7. All the other animals were euthanised at day 10. Change in individual mouse weight over time and viraemia data are shown in Supplementary Figure 1.

**Table 3:** Individual mouse outcomes.

**Individual**	**Survival time (days)**	**Clinical signs**
Mouse #1	10	Ruffled hair, reduced activity, hunching, weight loss
Mouse #2	8	Ruffled hair, reduced activity, hunching, ataxia, circling gait, ocular watery discharge
Mouse #3	7	Ruffled hair, reduced activity, hunching, laboured breathing, weight loss, tremors, circling gait, ocular watery discharge
Mouse #4	10	Ruffled hair, reduced activity, inflammation (oedematous swelling) at injection site, weight loss
Mouse #5	10	Ruffled hair, reduced activity, leg swelling

### 3.2. Histopathological findings

Following dissection only a small number of macroscopic lesions were observed. Some areas of discoloration could be seen in individual spleens and livers.

**Figure 1:**
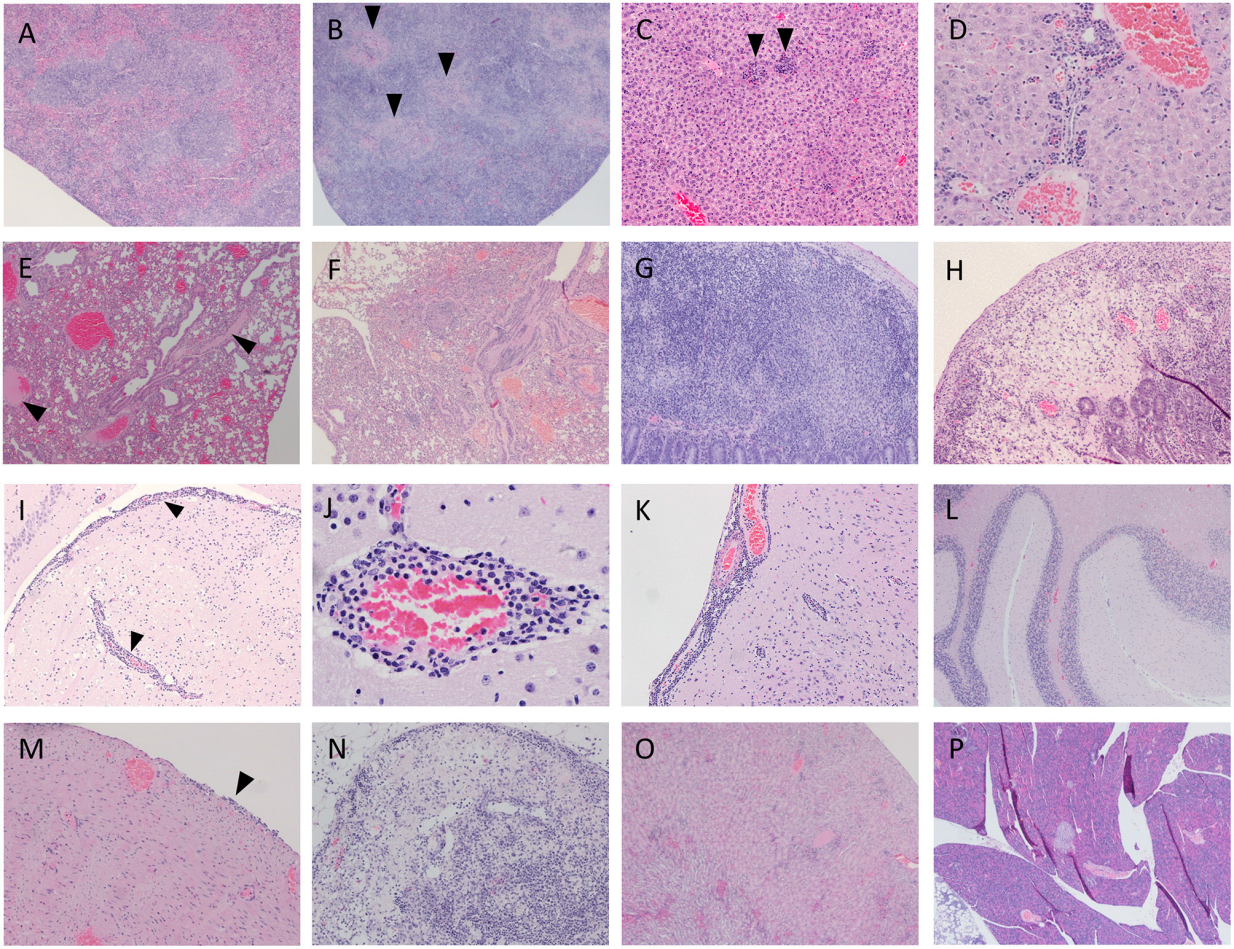
Representative images of infected spleen displaying both normal (**A**; mouse 2) and necrotic states (**B**, arrowhead; mouse 3). Liver tissue displays multifocal hepatitis (**C**, arrowhead; mouse 2) and inflammatory infiltrate around the portal triad (**D**; mouse 3). Lung tissue displays some oedema (**E**, arrowhead, mouse 1; and **F**; mouse 2) demonstrates interstitial pneumonia throughout tissue. In the gastrointestinal tract, Peyer's patches display lymphoid depletion after 7 days (**G**; mouse 3) and severe necrosis after 10 days (**H**; mouse 4). Cerebral tissue presented perivascular cuffing and meningeal inflammatory infiltrates (**I**, arrowhead; mouse 2 and **K**; mouse 3). Perivascular cuffing is also shown at higher magnification (**J**; mouse 3). Cerebellum tissue appeared consistently normal (**L**; mouse 2). One individual displayed signs of inflammation in the epicardium (**M**, arrowhead; mouse 3). Lymph nodes frequently displayed focal severe necrosis and lymphoid depletion (**N**, mouse 4). Mild inflammatory infiltrates were observed in kidney tissue (**O**; mouse 3). Pancreatic tissue was consistently normal (**P**; mouse 1).

A range of microscopic lesions of variable severity were detected within the organs of infected mice. Some spleen sections did not show any remarkable lesion while other animals showed mild to moderate necrosis (Figures 1A, B). All examined livers displayed either multi-focal necrotic hepatitis (Figure 1C), periportal inflammatory cell infiltration (Figure 1D), or both. Lung tissue consistently showed atelectasis, mild interstitial oedema with some areas of alveolar oedema and mild to moderate interstitial pneumonia (Figures 1E, F). The gastrointestinal tract tissue sections displayed mild inflammatory cell infiltration within the small intestinal villi and lymphoid depletion in the Peyer's patches (Figures 1G, H). Lesions in the brain consisted of perivascular cuffing and meningitis (Figures 1I–K) in the cerebrum, whereas cerebellum tissue was consistently normal (Figure 1L). Heart tissue sections did not show any remarkable changes apart from one animal showing mild inflammatory cell infiltration in the epicardium (Figure 1M); lymph nodes displayed lymphoid depletion and severe necrosis (Figure 1N). Kidney tissue did not show any remarkable lesion apart from mild inflammatory cell infiltrates in the cortex (Figure 1O) and pancreatic tissue appeared consistently healthy (Figure 1P). Results are summarised below in Table 4.

**Table 4:** Summary of individual mouse outcomes and histopathological findings.

**Individual**	**Survival Time (Days)**	**Organ**								
		**Spleen**	**Lungs**	**Liver**	**Brain (Cerebrum)**	**Heart**	**Kidney**	**Pancreas**	**Lymph Node**	**Intestine**
1	10	Mostly normal/ mild necrosis	Interstitial pneumonia, mild oedema	Periportal inflammation, foci of inflammation	Perivascular cuffing, meningitis	Congestion	Normal	Normal	-	-
2	8^†^	Mostly normal/mild necrosis	Interstitial pneumonia, mild oedema	Periportal inflammation, foci of inflammation	Perivascular cuffing, meningitis	Congestion	Normal	Normal	Normal	Normal
3	7^†^	Mild necrosis	Interstitial pneumonia, mild oedema	Periportal inflammation, foci of inflammation	Perivascular cuffing, meningitis	Epicardial inflammation, congestion	Normal	Normal	-	Lymphoid depletion in Peyer's Patch
4	10	Severe necrosis	Interstitial pneumonia, mild oedema	Periportal inflammation	Normal	Congestion	Normal	Normal	Necrosis	Necrosis in Peyer's Patch
5	10	-	-	-	-	-	-	-	-	-

^†^Euthanised at humane end point. – indicates tissue was unavailable for analysis (all tissues from mouse 5 were unavailable for analysis).

### 3.3. Immunofluorescent detection of viral antigen

**Figure 2:**
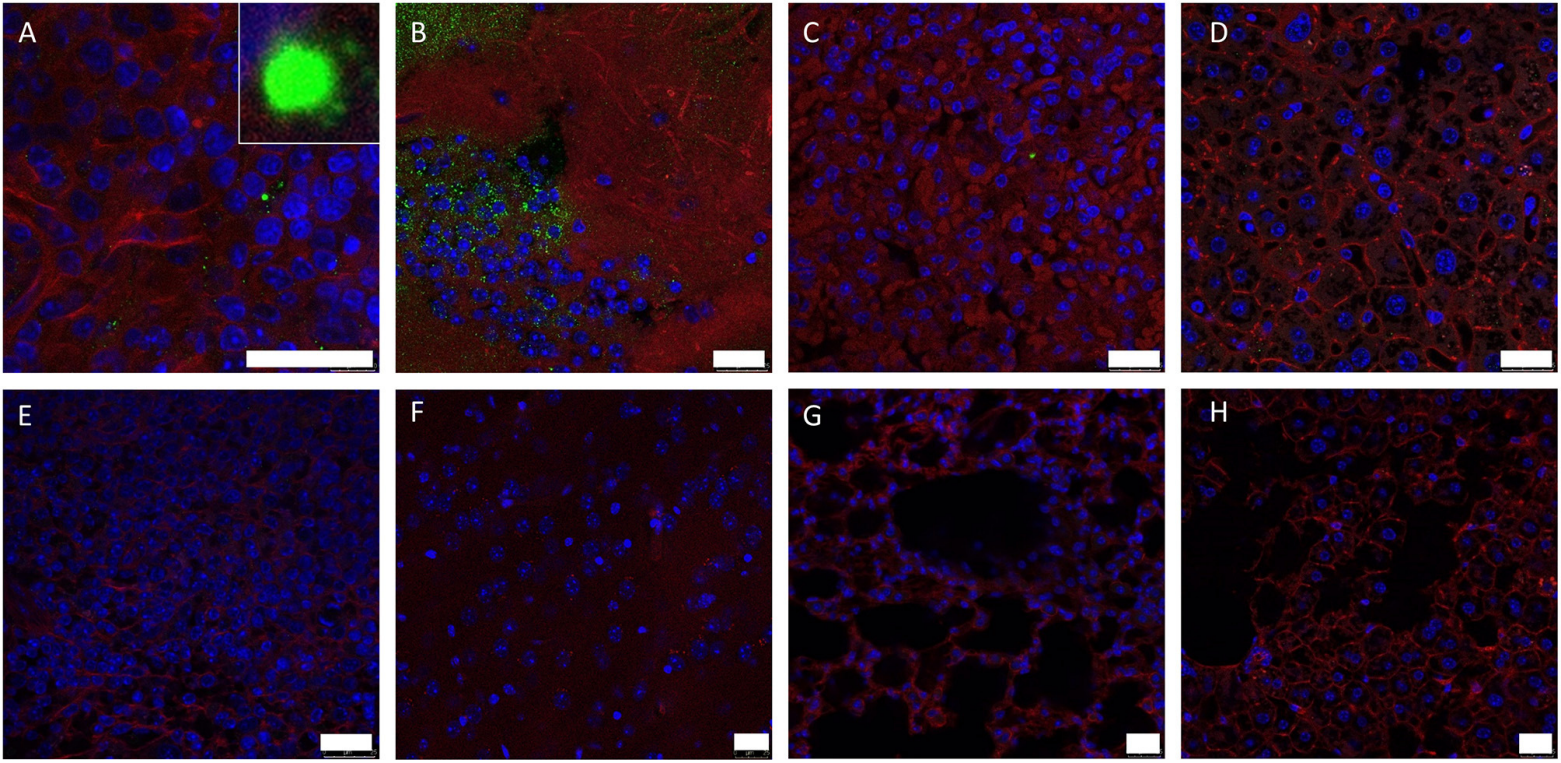
Splenic **(A, E)**, lung **(B, F)**, hepatic **(C, G)** and cerebral **(D, H)** tissue sections were stained with anti-AHSV VP7 mAb (green), phalloidin (red) and DAPI (blue) and analysed by confocal microscopy. Images from mouse 1 **(A–D)** and uninfected control mouse **(E–H)** are shown (no viral antigen was observed in uninfected control mouse tissue). High-magnification image of characteristic hexagonal VP7 crystal included as insert in **(A)**. Scale bars indicate 25 μm.

Discrete staining of viral protein VP7 could be detected in the spleen of infected IFNAR^−/−^ mouse 1 (Figure 2A), and very strong positive staining was observed within cerebral brain tissue (Figure 2B). Very low levels of staining were detected in the lung and liver (Figures 2C, D). At high magnification these positively stained foci had a geometric, regular structure 1–4μm in diameter and resembled the hexagonal VP7 crystals commonly associated with AHSV infection *in vitro* (30). No positive staining was observed in uninfected control tissue (Figures 2E–H).

### 3.4. Distribution of inflammatory cells within tissues

**Figure 3:**
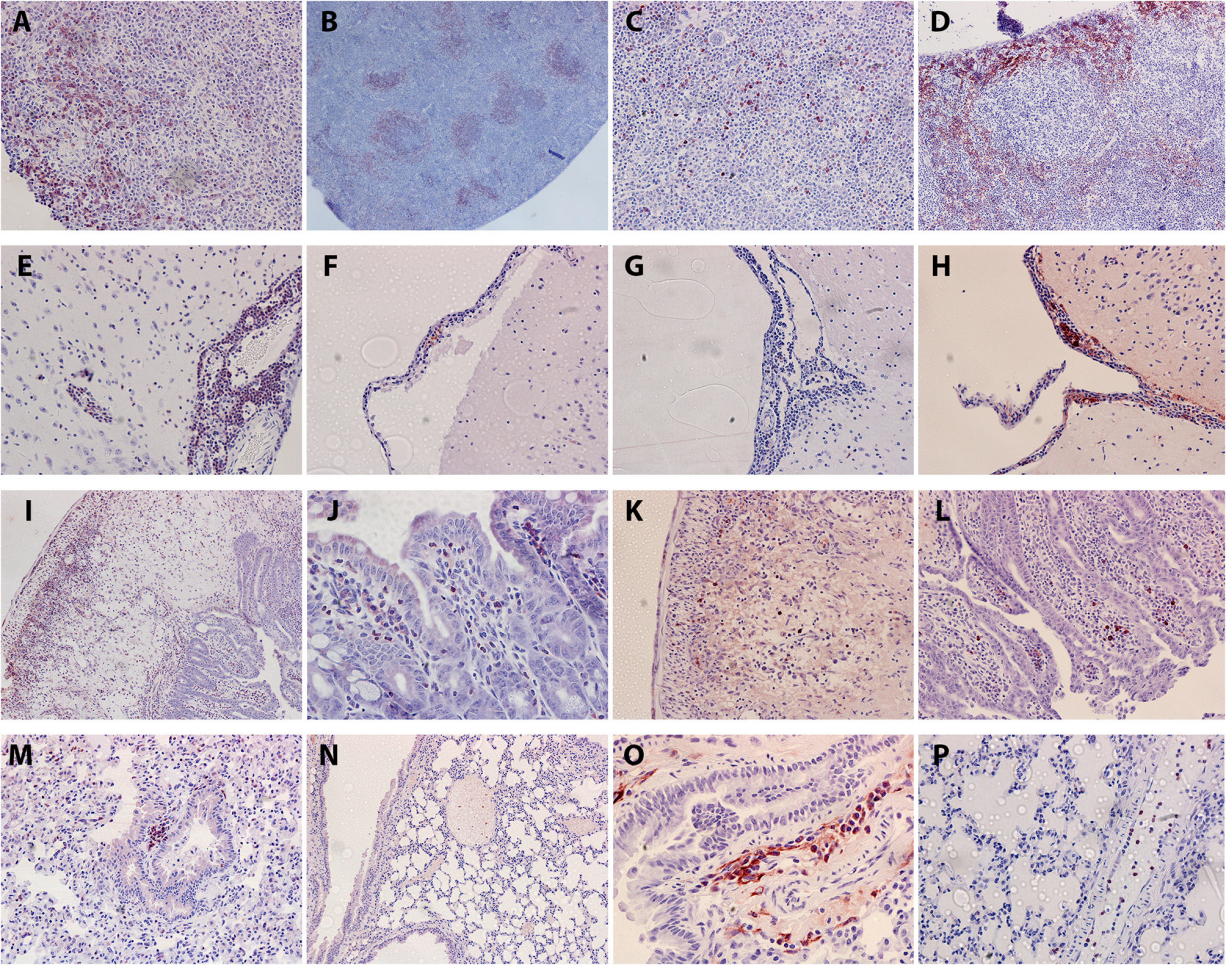
Representative examples of immunostaining within different murine organs (positive staining in red/brown). T cell staining **(A)**, B cell staining **(B)**, neutrophil staining **(C)** and macrophage staining **(D)** were all examined in the spleen. Within the brain, T cell staining **(E)**, neutrophil staining **(F)**, B cell staining **(G)** and macrophage staining **(H)** were all examined. In the intestine, T cell staining is visible in necrotic Peyer's patch **(I)** and surrounding villi **(J)**. Sparse macrophage staining is also present within necrotic Peyer's patch **(K)**. B cell staining in Peyer's patch and surrounding villi **(L)** is also sparse. Within the lung, staining was performed for T cells **(M)**, B cells **(N)**, macrophages **(O)** and neutrophils **(P)**.

Within the AHSV-4 infected splenic tissues, inflammatory cell distribution varied between those spleens that displayed few if any histopathological lesions, and those that displayed necrosis. In the less affected spleens, CD3-specific antibody staining revealed the presence of a moderate number of T-cells, mainly concentrated within the red pulp (Figure 3A). CD45R staining revealed large clusters of B cells within the white pulp (Figure 3B). Neutrophils appeared diffusely present throughout the tissue (Figure 3C). Strong positive staining for macrophages revealed their presence throughout the red pulp of the spleen (Figure 3D). In more necrotic spleens, staining levels for all cell types were reduced.

Within the brain, T cells were highly prominent within the perivascular cuffs and meningeal inflammatory infiltrates (Figure 3E). B cell and neutrophil staining was much sparser (Figures 3F, G) but macrophages were clearly detected within these regions (Figure 3H).

Intestine staining showed abundant T cells within the depleted Peyer's patches and the surrounding villi (Figures 3I, J). Some macrophage staining was also present in these regions (Figure 3K). Low levels of B cells could be detected in the villi near the Peyer's patches (Figure 3L), but no neutrophil staining was observed. Within the lungs, small patches of T cell infiltrates could infrequently be observed in the tissue, but no B cells could be detected (Figures 3M, N). Small infiltrations of macrophages (Figure 3O) into the tissue were also observed, but neutrophil staining was entirely restricted to blood vessels (Figure 3P). Heart tissue did not reveal any leucocyte staining within the cardiac muscle, but high levels of circulating T cells and neutrophils could be detected within the blood vessels or heart chambers. Moreover, some T cell infiltration was observed in the epicardium of the animal showing inflammatory cell infiltration with the H&E stain. In other organs staining was sparse; some macrophages were present in the inflammatory infiltrate in liver lesions, but none of the other cell types tested for were present. T cells were visible in the necrotic region of one particular lymph node; again, no other tested-for cell types were present. This immunohistochemical staining is summarised below in Table 5.

**Table 5:** Correlation of histopathology with immunohistochemical labelling in AHSV-4 infection (summary).

**Organ**	**Histopathology**	**Immunohistochemistry**			
		**CD3 (T cells)**	**CD45R (B cells)**	**Ly-6G (Neutrophils)**	**F4/80 (Macrophages)**
Spleen	Mild Necrosis	Moderate, diffuse positive staining	Low levels of diffuse, positive staining.	Moderate-to-strong diffuse positive staining	Moderate positive staining in red pulp
Liver	Periportal necrosis, multifocal inflammation	No staining	No staining	Circulating neutrophils	Strongly positive clustered infiltrates
Lung	Interstitial pneumonia, mild oedema	Localised positive staining	No staining	Circulating neutrophils	Localised positive staining
Heart	Normal. One animal showing epicardial infiltrates	Circulating T cells and epicardial infiltrates	Only background	Circulating neutrophils	No staining
Cerebrum	Perivascular cuffing, meningitis	Strong positive staining in meninges and cuffs	Minor background staining	Minor background staining	Strong positive staining in meninges
Cerebellum	Normal	No staining	No staining	No staining	No staining
Intestine	Tissue normal, Peyer's patches (PP) lymphoid depletion	Strong positive staining among villi, PP	Very low level staining among villi, PP	Low level staining among villi	Low-to-moderate staining in villi, PP
Lymph Node	Necrosis (variable)	Localised positive staining	No positive staining	No staining	Very low level staining in subcapsular sinus

In uninfected control spleens, B cells were abundant within the white pulp. T cells were also present in the white pulp, with some staining also present in the red pulp. Macrophages were strongly detected throughout the red pulp; neutrophils were also detected in the red pulp but more discretely. No significant staining of any kind was seen in uninfected brain tissue or in lung tissue. This staining is summarised below in Table 6 and shown in Supplementary Figure 2.

**Table 6:** Correlation of histopathology with immunohistochemical labelling in uninfected tissue.

**Organ**	**Histopathology**	**Immunohistochemistry**			
		**CD3 (T cells)**	**CD45R (B cells)**	**Ly-6G (Neutrophils)**	**F4/80 (Macrophages)**
Spleen	None	Strong positive staining, clusters throughout tissue	Very strong positive staining in white pulp	Moderate positive staining, clusters throughout tissue	Very strong positive staining throughout red pulp
Liver	None	No positive staining	No positive staining	No positive staining	No positive staining
Lung	None	No positive staining	No positive staining	No positive staining	No positive staining
Heart	None	*Staining not performed*	*Staining not performed*	*Staining not performed*	*Staining not performed*
Cerebrum	None	No positive staining	No positive staining	No positive staining	No positive staining
Cerebellum	None	*Staining not performed*	No positive staining	No positive staining	No positive staining
Intestine	None	*Staining not performed*	*Staining not performed*	*Staining not performed*	*Staining not performed*
Lymph Node	None	*Staining not performed*	*Staining not performed*	*Staining not performed*	*Staining not performed*

### 3.5. Image analysis quantification of leucocyte cell types

**Figure 4:**
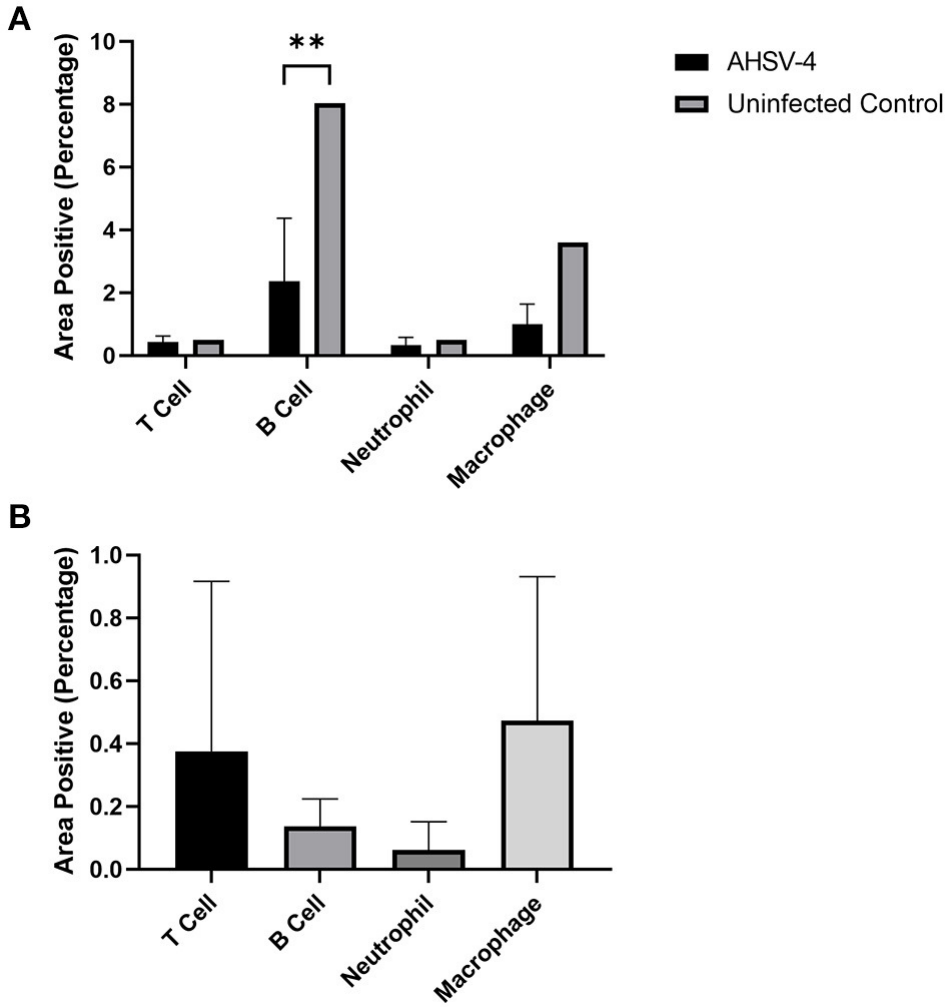
Image analysis was used to determine the % areas positive staining for different leucocytes in spleen (**A** – AHSV-4 infected and uninfected) and perivascular cuffs in the brain (**B** – AHSV-4 infected) tissues. The staining in tissue from an uninfected control mouse was compared to the mean staining in AHSV-4 infected mice using a 2-way ANOVA, where **indicates adjusted *p*-value < 0.01.

Image analysis shows that AHSV-4 infection was associated with an average 70.5% reduction in B cell staining and a 72.2% reduction in macrophage staining within the spleen, compared to the uninfected control tissue. Neutrophil and T cell numbers remained largely unchanged (Figure 4A). The level of staining was found to be significantly associated with both cell type and whether or not the tissue is infected (*p*-values of < 0.001 and 0.004 respectively). A subsequent Tukey's pairwise test revealed a statistically significant difference between B cell staining in infected spleens compared to the uninfected splenic tissue (adjusted *p*-value 0.008).

In the perivascular cuffing lesions observed in the brain, macrophages were the most frequently detected leucocyte, closely followed by T cells. B cells were also present, but almost no neutrophils were detected (Figure 4B). A one-way ANOVA test revealed the differences between these cell types were non-significant (*p* = 0.441). A negative control was not included, as the uninfected tissue lacked the lesions (swollen meninges with inflammatory infiltrates) to compare.

## 4. Discussion

The study of immunobiology and pathogenesis of AHSV is complicated due to logistical, financial, ethical and bio-safety factors associated with performing experimental infections of horses in BSL-3 facilities. The IFNAR^−/−^ animal model has been successfully used on many occasions and in this study we aimed to further characterise the pathology of AHSV infection in this model.

Other mouse models have been used to study AHSV *in vivo* in the past. Earlier studies used Balb/C mice (7), but in these experiments the virus had low pathogenicity unless injected intranasally. Previous IFNAR^−/−^ mice (31, 32) studies reliably reproduced a disease that shared some pathological similarities to AHS in horses (20), namely similar lesions in the spleen, lung and liver. AHSV-infected mice sometimes display neurological clinical signs, which are less commonly observed in horses.

In this experiment, histopathological analysis showed some minor lesions in the spleen. This is an expected result, as the virus is known to target spleens in the mouse host. AHSV also targets the spleen in the natural equine host, particularly the large mononuclear cells of the red pulp (16). However, the severity of the lesions was less than expected, with some individual spleens appearing completely healthy at the time of euthanasia. There was no direct correlation with spleen necrosis severity and time of death, with severe necrosis being observed in mouse 4 (euthanised 10 days post infection). A previous study with this virus strain showed that the spleen lesions of some AHSV-infected mice, especially those infected with a dose of 10^5.8^ pfu/mouse, presented severe lymphoid depletion with the red and white pulp becoming indistinguishable, and an abundant tissue necrosis (20). In the present study, this level of severity was not observed in any of the challenged animals, the most notable lesion being the presence of minor necrosis and presence of foci of amyloid degeneration, which were more consistent with the IFNAR^−/−^ mice infected with 10^4^ pfu/mouse in the study described above. In this experiment we also observed a significant depletion of B cells within the spleen, and a smaller reduction of macrophage numbers. A reduction in macrophages is more expected, as monocyte-macrophages are a target for the virus in the equine host (13) and this may also be the case in the murine host. The significant reduction in B-cell numbers observed could be due to direct infection and lysis by AHSV – previous studies are ambiguous on whether lymphocytes are targeted during infection, with one study finding AHSV antigen in large mononuclear cells but being unable to distinguish whether they were monocytes or lymphocytes (17). However, the observed B cell depletion could also be due to the generalised necrosis seen in infected spleens, or a dysfunctional immune response. Lymphoid depletion is a common feature of AHSV infection in horses (13, 33), and there is also evidence a dysfunctional immune response is partially responsible for the pathology observed in equine hosts (19, 34), but more research is needed. Immunofluorescence results show only limited presence of the virus in the spleen. This is consistent with the H/E staining revealing a range of non-severe lesions, from normal condition to mild necrosis.

Lesions in the liver, characterised by peri-portal inflammatory infiltration and focal necrosis, and in the lungs, characterised by oedema and interstitial pneumonia, were consistent with previous findings (20). The brain lesions (meningitis, perivascular cuffing) observed in this study are also consistent with previous experimental infections of IFNAR^−/−^ mice (20). Neurological clinical signs of AHS in horses are rarely reported and almost exclusively observed in animals vaccinated with AHSV-5 “neurotropic” attenuated vaccines, prepared by serial passage in neonatal mouse brains (35). It might be possible that field AHSV strains have the potential to cause central nervous system pathology, depending on additional factors such as virus strain, virus infectious dose and viraemia levels, and that this has gone unnoticed because of the predominant cardio-respiratory clinical signs. However, there is very limited evidence in the literature of AHSV neuropathology in horses. It is important to note that the involvement of neuronal damage as a result of viral infection has not yet been demonstrated. In this study, despite substantial presence of AHSV antigen in the brain, as observed by immunofluorescence, we could not unequivocally determine the cell types harbouring the virus as immunohistochemistry procedures to detect viral antigens in the brain neurons could not be performed reliably. What was very evident, however, was the presence of abundant inflammatory cells in perivascular spaces and meninges, which can explain the neurological clinical manifestations of AHSV infection in the IFNAR^−/−^ mice. In addition, the abundance of T-cells and macrophages in the inflammatory infiltrates in the brains (Figure 3E) is characteristic of AHSV infection in this animal model and typical of viral meningitis (36, 37). T cells target virally infected cells for destruction, and in this instance are likely targeting infected endothelial cells lining the capillaries. The combination of viral infection and programmed cell death induced by T cells are the proposed basis of the neuronal tissue damage and may explain the neurological signs observed.

Very little viral presence being detected in the liver and lung is more surprising. Histopathological results showed mild to severe lesions in all the livers examined, and severe pneumonia and oedema in the lungs, but very little viral antigen was detected by immunofluorescence. Other studies in the equine host have also shown severe pulmonary tissue damage seemingly at odds with low levels of detectable viral presence (15, 38). It is possible that the severity of the lesions may partly be due to a dysfunctional immuno-pathological component. Although IFNAR^−/−^ mice lack a functional IFN-α receptor, they are not immunodeficient otherwise, and may develop severe immunopathology should an overwhelming and dysfunctional immune response occur after infection. Bluetongue virus (closely related to African Horse Sickness virus) pathogenesis seems to have an immunopathogenic component (39–41). Whether a similar mechanism occurs in AHSV remains to be demonstrated. Alternative mechanisms of lung tissue damage include viral lysis of infected endothelial cells resulting in excess fluid leakage and oedema. Previous studies in horses suggest that direct viral infection of endothelial cells plays a role in the severe pulmonary oedema seen, but other mechanisms are likely also involved (38), possibly involving the infection of monocyte-macrophages in the lung (14). Alternatively, the lung pathology may be due to infection or activation of pulmonary interstitial macrophages, in turn triggering a pro-inflammatory response associated with microvascular leakage, which has been observed in African Swine Fever virus infection (42). More research is needed to elucidate the underlying cause of the severe lung pathology.

This experiment showed the Peyer's patches becoming highly necrotic following infection. However, T Cells, a normal component of the tissue, could still be detected (Figure 3I). Macrophages could also be detected, perhaps present to phagocytose debris from the necrosis. Macrophages in the liver (Figure 3M) may again be playing a role in clearing up cell lysis debris, or the stain may have labelled Kupffer cells. T cells can be found in lymph nodes under normal circumstances, and are still present even surrounded by necrosis (Figure 3N).

In conclusion, we have gained further knowledge about the histopathological characteristics of the IFNAR^−/−^ mouse model of AHSV. We have also used various techniques, including histopathology, immunohistochemistry and immunofluorescence to achieve insights into the viral tropism and the impact of the virus on the host immune system. We showed that the main target organs of AHSV in this model, and tissue lesions (severe lung lesions with surprisingly low viral presence, or lesions in the spleen and liver) are the same as in the natural AHSV infection in the target species, with the exception of the brain. Future research aiming at identifying molecular determinants of virulence and pathogenicity could use this well-characterised animal model.

## Data availability statement

The original contributions presented in the study are included in the article/Supplementary material, further inquiries can be directed to the corresponding author.

## Ethics statement

The animal study was reviewed and approved by the Animal Welfare and Ethical Review Board (AWERB) of The Pirbright Institute.

## Author contributions

JC-O and FJS conceived, designed, and performed the animal experiment. LJ and PH performed analytical experiments. LJ, JC-O, and FJS wrote the paper. All authors contributed to the article and approved the submitted version.

## Supplementary material

Supplementary Figure 1 RT-PCR ct values taken from individual mouse blood, on various days post AHSV-4 challenge **(A)**. The percentage change in individual mouse weight (from day 0) is also depicted **(B)**.

Supplementary Figure 2 Immunohistochemical staining of uninfected tissues. **(A–D)** show splenic tissue, **(E–H)** display cerebrum tissue, and **(I–L)** display pulmonary tissue. B cell staining is shown in images **(A, E, I)**; macrophage staining is shown in images **(B, F, J)**; neutrophil staining is shown in images **(C, G, K)**; T cell staining is shown in images **(D, H, L)**.
