# Supplementary figures and images for: Pathological features of African horse sickness virus infection in IFNAR−/− mice
Source: Front Vet Sci. 2023 Mar 30;10:1114240. doi: 10.3389/fvets.2023.1114240 (PMC10098166; doi:10.3389/fvets.2023.1114240)

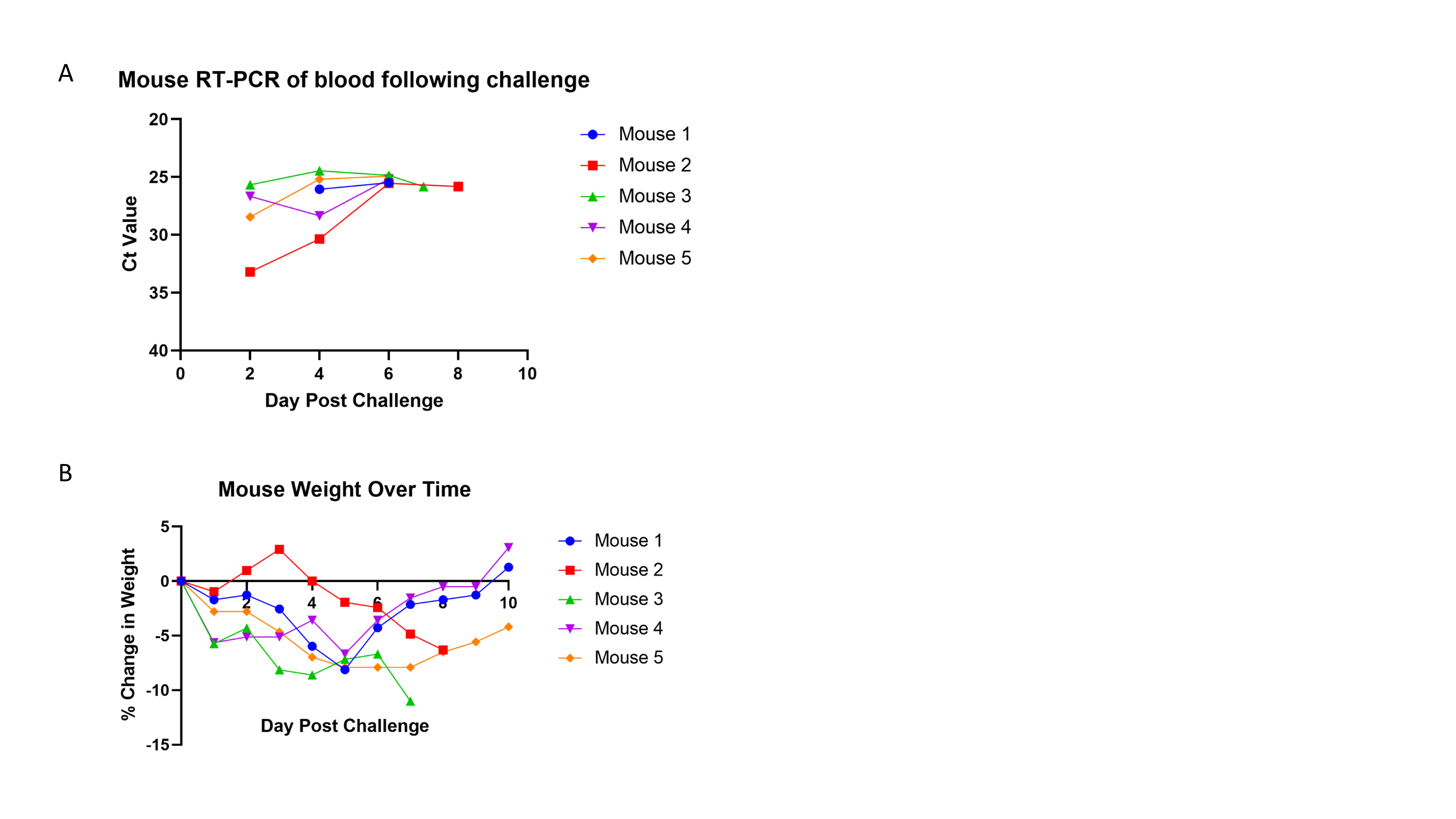

Supplement: Supplementary Figure 1 — RT-PCR ct values taken from individual mouse blood, on various days post AHSV-4 challenge (A). The percentage change in individual mouse weight (from day 0) is also depicted (B). [file Image_1.TIF]

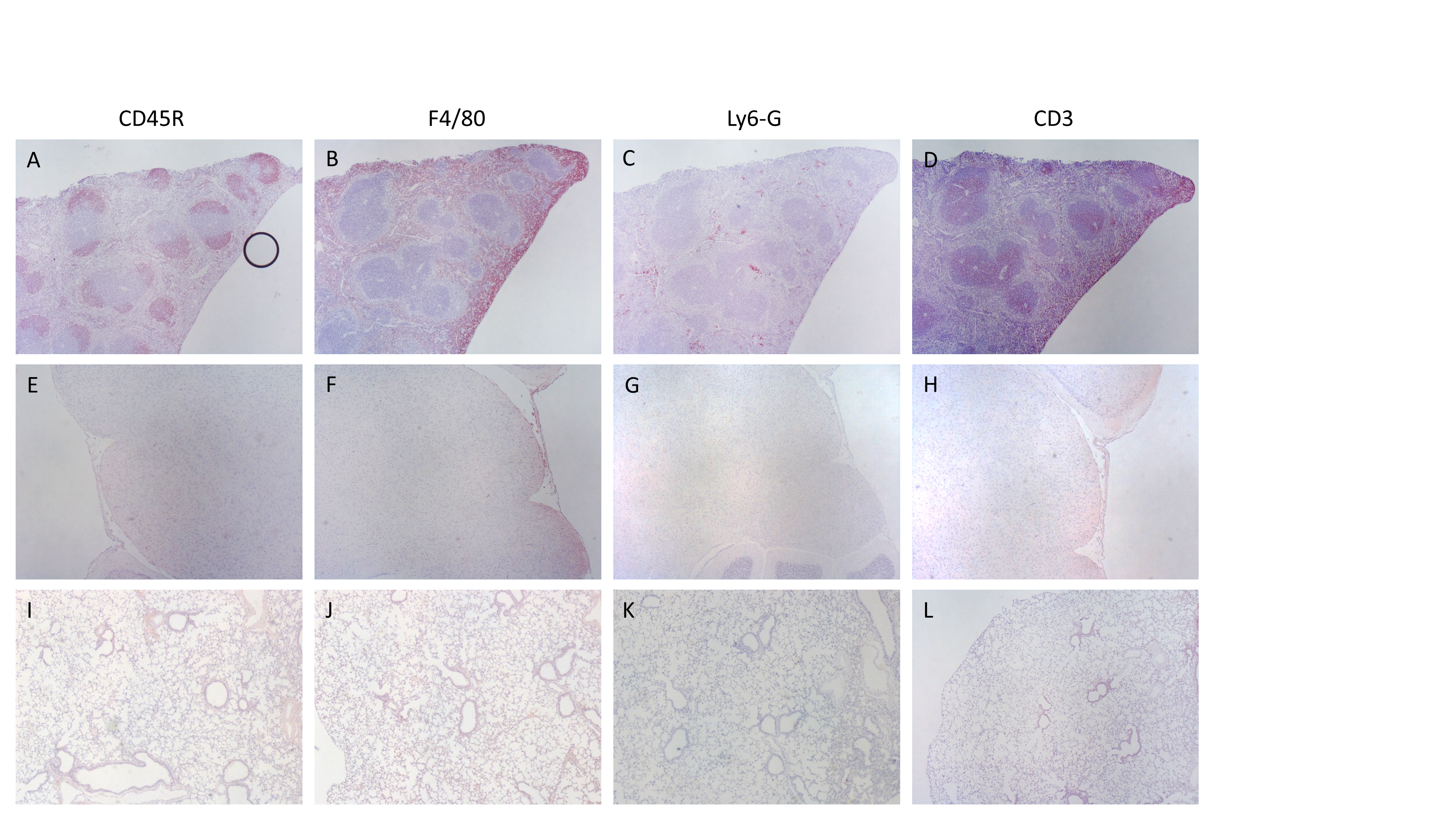

Supplement: Supplementary Figure 2 — Immunohistochemical staining of uninfected tissues. (A–D) show splenic tissue, (E–H) display cerebrum tissue, and (I–L) display pulmonary tissue. B cell staining is shown in images (A, E, I); macrophage staining is shown in images (B, F, J); neutrophil staining is shown in images (C, G, K); T cell staining is shown in images (D, H, L). [file Image_2.TIF]
